# Formulation of Antioxidant Composites by Controlled Heteroaggregation of Cerium Oxide and Manganese Oxide Nanozymes

**DOI:** 10.1021/acs.jpcc.3c03964

**Published:** 2023-08-18

**Authors:** Nizar B. Alsharif, Dániel Viczián, Aleksandra Szcześ, Istvan Szilagyi

**Affiliations:** † MTA-SZTE Lendület Biocolloids Research Group, Department of Physical Chemistry and Materials Science, Interdisciplinary Research Center, University of Szeged, H-6720 Szeged, Hungary; ‡ Department of Interfacial Phenomena, Institute of Chemical Sciences, Faculty of Chemistry, Maria Curie-Skłodowska University, PL-20031 Lublin, Poland

## Abstract

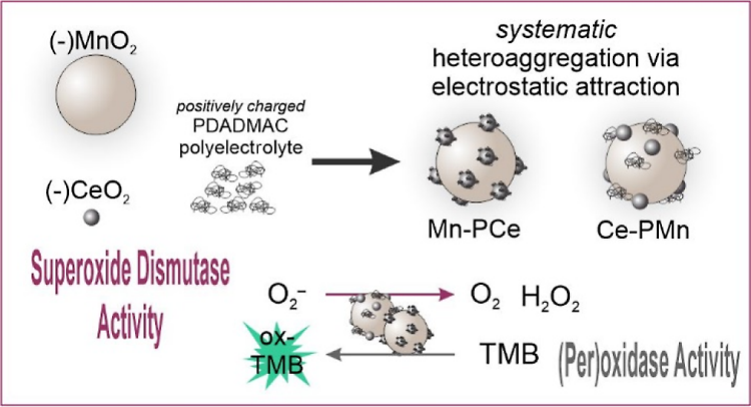

Antioxidant composites based on nanozymes [manganese oxide microflakes (MnO_2_ MFs) and cerium oxide nanoparticles (CeO_2_ NPs)] were formulated by controlled heteroaggregation. The interparticle attraction via electrostatic forces was systematically tuned with surface functionalization by the poly(diallyldimethyl chloride) (PDADMAC) polyelectrolyte. The PDADMAC-coated MnO_2_ MFs (PMn) were heteroaggregated with oppositely charged CeO_2_ NPs to generate the Ce–PMn composite, while the PDADMAC-functionalized CeO_2_ NPs (PCe) were immobilized onto bare MnO_2_ MFs, resulting in the Mn–PCe composite. Both the adsorption of PDADMAC and the self-assembly of oppositely charged particles resulted in charge neutralization and charge reversal at appropriately high doses. The interparticle force regimes, the aggregation states, and the physicochemical properties of the relevant dispersions were also highly dependent on the dose of PDADMAC, as well as that of PDADMAC-functionalized metal oxides (PMO) enabling the fine-tuning and control of colloidal stability. The individual enzyme-like activity of either metal oxide was not compromised by PDADMAC adsorption and/or heteroaggregation, leading to the formation of broad-spectrum antioxidant composites exhibiting multiple enzyme-like activities such as superoxide dismutase, oxidase, and peroxidase-type functions. The low cost and ease of preparation, as well as controllable colloidal properties render such composites potential enzyme mimicking agents in various industrial fields, where processable antioxidant systems are needed.

## Introduction

Native enzymes have remarkable catalytic properties of significant importance in biological and industrial systems.^1,2^ Beside excellent catalytic rates, enzymes possess high selectivity as a result of intricate chemical structures.^2−4^ Nevertheless, such complexity in structures is associated with several inherent disadvantages such as loss of activity at non-optimal pH and temperature ranges, as well as in the presence of inhibitors. For example, the optimal temperature and pH range of the superoxide dismutase (SOD) enzyme is 30 °C and 6.0–7.0, respectively. However, a near total loss of activity is observed, when the temperature is 70 °C or 10 °C, or when pH is changed to 3.0.^5^ Similarly, the peroxidase enzyme is characterized by an optimal activity at pH 4.8, which rapidly deteriorates when the pH is changed to 3.0 or 5.5. In addition, the optimal temperature for peroxidase is 35 °C, while a total loss of activity can be observed at 60 °C.^6^ Hence, undesignated conditions adversely alter the structure of the comprising proteins and eventually cause a permanent loss of the catalytic activity. Moreover, the production and purification of enzymes are highly complicated and time-consuming processes, which has rendered them rather expensive materials.^4,7^

Enzymes have been heavily used in various fields such as food, pharmaceutical, cosmetic, and textile industries, and hence, there has been a strong demand for versatile alternatives that function at harsh conditions and yet possess similar catalytic properties as the native biocatalysts.^8,9^ While various chemical structures have exhibited enzyme-like potential such as cyclodextrin, molecularly imprinted polymers, and antibodies;^10,11^ enzymatically active nanomaterials (nanozymes) have emerged as optimal artificial enzymes due to easy and low-cost preparation, high stability, as well as tuneable physicochemical properties.^12−14^ In addition, the enzymatic activities are typically preserved at pH or temperature conditions that are well outside the optimal window of the corresponding native enzymes.^15^ Among the important mimicked enzyme classes are the oxidoreductase enzymes, which help regulate reactive oxygen species (ROS) such as superoxide radical (^•^O_2_^–^), hydrogen peroxide (H_2_O_2_), and hydroxyl radical (^•^OH). These enzymes include SOD, catalase, oxidase, and peroxidase. Numerous materials of various structures and compositions have been found to mimic such enzymes including metallic nanoparticles (Ag, Au, and Pd),^16−18^ metal oxides (Co_3_O_4_, CeO_2_, and V_2_O_5_),^19−23^ metal chalcogenides (FeS, MoS_2_, and WS_2_),^24−27^ clays,^28,29^ carbon materials (fullerenes and carbon nanotubes),^30^ biopolymers,^31,32^ and metal–organic frameworks.^33^ The surface functionalities and parameters of nanoparticles such as metal oxides often result in the aggregation of the bare and primary particles into clusters of various sizes, depending on the experimental conditions in the system such as ionic strength, the presence of polyelectrolytes, and the nature of the solvents.^34−36^ In addition, many metal oxide particles are characterized by an isoelectric point (IEP),^37,38^ at which the particles have no surface charge and tend to undergo heavy aggregation that results in the loss of the surface area, which could lead to a significant reduction of the enzymatic activity.

Our previous studies have detailed the SOD-, catalase-, and peroxidase-like activities of manganese oxide microflakes (MnO_2_ MFs) and cerium oxide nanoparticles (CeO_2_ NPs) along with a thorough characterization of the colloidal properties of both particles.^15,39^ To stabilize such highly aggregating systems, the MnO_2_ MFs and CeO_2_ NPs were immobilized onto sulfate-functionalized polystyrene latex (SL) microspheres as carriers to preserve their catalytic surface. Since both metal oxides are biocompatible, the potential of MnO_2_ MFs and CeO_2_ NPs in biomedical applications mandates the choice of a biocompatible carrier due to the potential toxicity effect of the polystyrene latex particle at the cellular level.^40,41^ Such engineered composites possess various advantageous features and have been heavily explored for several applications like catalysis,^42^ biomedical treatment,^43^ sensing,^44^ water purification,^45^ drug delivery,^46^ and energy storage.^47^ While the composites can be prepared via sol–gel or hydrothermal preparation routes,^48,49^ the heteroaggregation of pre-fabricated particles can also be utilized to formulate highly functional hybrid materials. In this way, systematic mixing of the component dispersions in the proper ratios allows the control of the final properties including surface charge and size.^50^

**Scheme 1:**
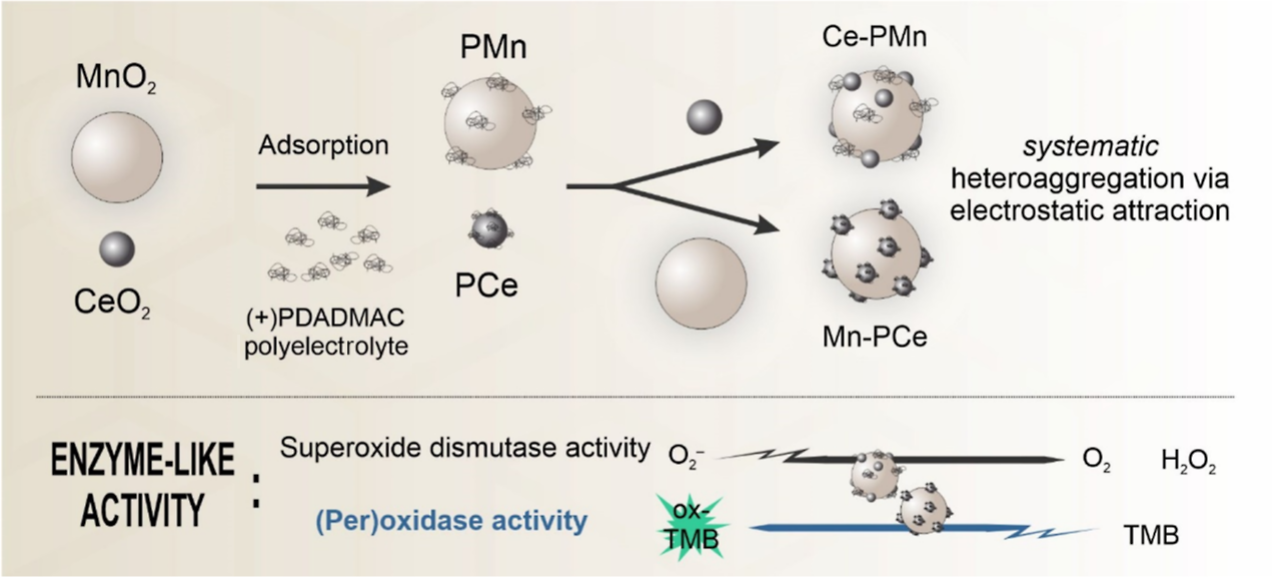
Illustration of the Systematic Heteroaggregation between Bare Metal Oxides (MnO_2_ and CeO_2_) and PDADMAC-Functionalized Metal Oxides (PCe and PMn) to Formulate Ce–PMn and Mn–PCe Composites under the Influence of Electrostatic Forces The formulated composites exhibited oxidase, peroxidase, and SOD activity.

In the present report, our previous studies on MnO_2_ MFs^15^ and CeO_2_ NPs^39^ are expanded. Two antioxidant composites were formulated by systematic heteroaggregation of the MnO_2_ MFs and CeO_2_ NPs. As shown in Scheme 1, the surfaces of both metal oxides were initially modified with the PDADMAC polyelectrolyte to obtain positively charged PCe and PMn (PDADMAC-functionalized metal oxides). Then, PMn was heteroaggregated with oppositely charged CeO_2_ NPs to generate Ce–PMn, while PCe was heteroaggregated with bare MnO_2_ MFs to obtain Mn–PCe composites. Electron microscopy and light scattering techniques were used to probe the structural and colloidal properties during both the surface modification and heteroaggregation to obtain highly stable PDADMAC-functionalized particles and fine dispersions of the hybrid antioxidant particles. Standardized enzymatic assays were performed to probe enzymatic activities as well as any interaction between the metal oxides that could interfere with the individual activities.

## Experimental Methods

### Materials

Xanthine (99%) was obtained from Alfa Aesar, and nitro blue tetrazolium chloride (NBT, 90%) was purchased from Acros Organics. Xanthine oxidase (0.4–1.0 units/mg protein, lyophilized powder), 3,3′,5,5′-tetramethylbenzidine (TMB) (≥99%), and PDADMAC (200–350 kg/mol, 20 wt %) were purchased from Sigma-Aldrich. NaCH_3_COO·3H_2_O (≥99.5%) and glacial acetic acid (Ph.Eur./USP), obtained from VWR, were used to prepare acetate buffer (pH 4.0). Phosphate buffer (pH 7.0) was prepared using NaH_2_PO_4_ (99%, anhydrous) and Na_2_HPO_4_ (≥99%, GPR RECTAPUR) purchased from Acros Organics and VWR, respectively. Tris(hydroxymethyl) aminomethane (TRIS) (AnalaR NORMAPUR), bought from VWR, was used to prepare TRIS–HCl buffer (pH 9.0). Dimethyl sulfoxide (AnalaR NORMAPUR), HCl (37 w/w %, AnalaR NORMAPUR), NaOH (≥99.3%, AnalaR NORMAPUR), H_2_O_2_ (30% w/w), and NaCl (99.9%) were obtained from VWR. The glass and plasticware were cleaned using the Hellmanex III reagent, bought from Hellma. Ultrapure water was obtained from the Adrona water purification system (Bio-Science Kft) and was further filtered using polyvinyl difluoride-based syringe filters (0.1 μm, Millex-VV).

### Light Scattering

The colloidal stability of the individual materials and the composites was assessed at 25.0 ± 0.2 °C by light scattering techniques to obtain the zeta potential values and aggregation rates. The electrophoretic mobilities were recorded using the Litesizer 500 (Anton Paar) device (a 658 nm laser source and 200 V voltage were applied) and omega-shaped plastic cuvettes (Anton Paar). In addition, the initially recorded electrophoretic mobilities were converted to zeta potentials (ζ) using the Helmholtz–Smoluchowski equation^51^
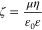
1where μ is the electrophoretic mobility, η is the medium’s dynamic viscosity, ε_0_ is the permittivity of the vacuum, and ε is the dielectric constant of the medium. The reported zeta potential values were the average of 5–8 runs.

The aggregation rates were estimated with dynamic light scattering (DLS) using an ALV-NIBS/HPPS particle sizer, equipped with a 632.8 nm wavelength laser source, in polystyrene (PS) disposable cuvettes. The intensity of the light was collected at a scattering angle of 173°, and the resulting correlation function was analyzed using the cumulant fit to obtain the translational diffusion coefficients, and hence, the hydrodynamic radii using the Stokes–Einstein equation.^52^ In time-resolved DLS, the change in hydrodynamic radius (*R*_h_) was recorded over time (30–100 measurement points), and for a given sample, the apparent aggregation rate constant (*k*_app_) was obtained as follows^53^
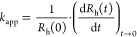
2where *R*_h_(0) is the hydrodynamic radius of non-aggregated monomer particles and  is the slope of the linear fit of the hydrodynamic radii-time data determined for the sample under study. The stability ratio (*W*) was calculated using the following expression^54^
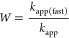
3where *k*_app_ is the apparent aggregation rate constant for the sample in question and *k*_app(fast)_ is the apparent aggregation rate constant for a rapidly aggregating dispersion, typically at 1.0 M ionic strength, where electrostatic repulsive forces are largely ineffective due to charge screening. The stability ratio is a measure of colloidal stability and is directly proportional to the stability of a colloidal dispersion, i.e., inversely proportional to *k*_app_. Hence, stable dispersions have high stability ratios, while unstable systems, which undergo diffusion-controlled particle aggregation, result in stability ratio values near unity.^55^

For previously characterized CeO_2_ NPs and MnO_2_ MFs, the zeta potentials and aggregating rates were obtained to probe the effect of PDADMAC dose on the charge of both particles at pH 9.0 and 1.0 mM ionic strength, the latter one was adjusted by NaCl. The goal of the PDADMAC coating was to obtain positive charge on one of the particles to induce heteroaggregation via electrostatic forces (see Scheme 1). In each case, the metal oxide concentration was fixed at 100 mg/L while the dose (mg PDAMDAC/g metal oxide) of the PDADMAC was varied. In both systems, the aggregation rate was measured immediately after the addition of the PDADMAC, while the zeta potential was obtained after equilibrating the samples for 2 h. The optimal PDADMAC dose, as will be detailed later, will give rise to highly charged and PDADMAC-saturated metal oxides (PMO), namely, MnO_2_ MFs (PMn) and CeO_2_ NPs (PCe). After the selection of the proper doses of PDADMAC-saturated PMn and PCe, the aggregation rates and zeta potentials were obtained during the heteroaggregation of bare metal oxide with different PMOs. The first system (Mn–PCe) was formulated by the addition, to a MnO_2_ MFs dispersion (100 mg/L), of different doses of PCe (in mg PCe/g MnO_2_ MFs) at pH 9.0 and 1.0 mM ionic strength. Inversely, the second system (Ce–PMn) was prepared by the heteroaggregation between a given concentration (50 mg/L) of CeO_2_ NPs and different doses of PMn (in mg PMn/g CeO_2_ NPs) at pH 9.0 and 1.0 mM ionic strength. In both systems, the aggregation rate was measured immediately after the addition of PMO dose, while the zeta potential was obtained after the samples were equilibrated for 2 h.

### Scanning Transmission Electron Microscopy

The samples were grinded to fine powders in an agate mortar. The obtained powders of each sample were poured with 99.8% ethanol (Avantor) to form a slurry, which, subsequently, were inserted into an ultrasonic homogenizer for 10 s. Then, slurry containing the samples were pipetted and supported on a 300 mesh copper grid covered with lacey formvar and stabilized with carbon (Ted Pella) and left on a filter paper for ethanol evaporation. The samples deposited on the grid were inserted into a single-tilt holder and moved to the electron microscope. The high-resolution electron microscope Titan G2 60–300 kV (FEI) was used to display the materials investigated. The microscopy studies were carried out at an accelerating voltage of the electron beam equal to 300 kV for the materials used. The elements mapping was carried out in the scanning transmission electron microscopy (STEM) mode by collecting point by point energy-dispersive spectroscopy (EDS) data of each of the corresponding pixels in the map. The collected maps were presented in the form of a matrix of colored pixels with the intensity corresponding to the amount of the element. Phase separation was performed with the fast Fourier-transform (FFT) generated from high-resolution TEM (HR-TEM) images of the samples.

### Superoxide Dismutase Activity

The SOD activity of the composite systems was assessed using the Fridovich assay.^56^ The SOD enzyme catalyzes the dismutation of superoxide radicals into H_2_O_2_ and water. Hence, in this assay, the superoxide radicals are produced via the oxidation of xanthine molecules by xanthine oxidase enzyme. The generated superoxide radicals react with the yellow indicator compound, NBT, to produce a purple product, called formazan. The reduction of NBT results in a strong absorption peak at 565 nm. In the presence of SOD mimicking composites, however, superoxide radicals are scavenged and hence, the NBT-superoxide radical reaction is inhibited, which leads to lower color intensity of formazan, i.e., smaller absorbance values. In a typical measurement, only the composite concentration was changed between 0 and 10 mg/L, while the concentration of the phosphate buffer (pH 7.0) was kept at 50 mM. In each of the final samples, a given volume of the composite was added to 1500 μL xanthine (0.4 mM), 100 μL NBT (3.0 mM), followed by the addition of a portion of phosphate buffer to get a 2700 μL sample. To initiate the reaction, 300 μL of xanthine oxidase (1.5 g/L) was added and then, immediately vortexed for 5 s. The increase in the absorbance over time was recorded for 6 min at 565 nm wavelength with a Genesys 10S spectrophotometer. In addition, the absorbance–time plots were measured for blank samples, in which the composite materials were absent. In these samples, all components were added and topped up with an additional volume of phosphate buffer to maintain similar concentrations in the final blank samples. The inhibition (*I*) of the NBT-superoxide radical reaction was calculated using the following expression
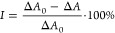
4where Δ*A* is the change in absorbance during the 6 min measurement time for the sample under study, and Δ*A*_0_ is the averaged change in the absorbance for the blank samples (eight of them were run) after 6 min. The inhibition was plotted against the corresponding composite concentrations and in such plots 0% indicated no radical scavenge, while 100% referred to complete elimination of the superoxide radicals by the nanozymes. Note that experiments performed without the presence of any nanozymes served as the control. The concentration of the composite, at which the inhibition was 50%, was termed as the IC_50_ value. During the assays, although light scattering by the particles might contribute to the absorbance, the scattering contribution was eliminated by reporting the relative absorbance change.

### Peroxidase Activity

The TMB assay was used to assess the peroxidase activity.^57^ The peroxidase enzyme breaks down peroxide substrates, which results in the production of radicals that act on a second substrate. This is typically accompanied by a color change that can be quantitatively studied via spectrophotometry. In the presence of H_2_O_2_ and peroxidase mimicking materials, the colorless TMB substrate is oxidized to a blue product (denoted as oxidized TMB) characterized by a strong absorption peak at 652 nm. The peroxidase activity of bare (MnO_2_ MFs and CeO_2_ NPs) and functionalized nanozymes (PMn and PCe), as well as Mn–PCe and Ce–PMn hybrids was assessed at acidic, neutral, and alkaline pH conditions using acetate (pH 4.0), phosphate (pH 7.0), and Tris-HCl (pH 9.0) buffers, respectively. In addition, the TMB was dissolved in DMSO due to its poor solubility in water. In the final samples (2.0 mL), the following concentrations were set: TMB (0.5 mM), buffer (50 mM), H_2_O_2_ (15.0 mM) and nanozyme (15.0 mg/L). The absorption spectrum of the sample after the prospective enzymatic reaction was recorded.

### Oxidase Activity

In the oxidase assay, the colorless TMB is directly oxidized by the nanozymes (without the involvement of H_2_O_2_), and the blue color of the oxidized TMB is quantitatively observed by recording the absorption spectra and observing the newly appearing peak at 652 nm after the anticipated enzymatic activity.^58^ Like the peroxidase assay, the oxidase activity of bare and functionalized nanozymes, as well as Mn–PCe and Ce–PMn was assessed at acidic, neutral, and alkaline pH conditions using acetate (pH 4.0), phosphate (pH 7.0), and Tris-HCl (pH 9.0) buffers, respectively. Here, the TMB was also dissolved in DMSO. In the final samples (2.0 mL), the concentrations of TMB, buffer, and nanozymes were set at 0.5 mM, 50 mM, and 15.0 mg/L, respectively. The visible absorption spectra of the samples were recorded after the reaction terminated.

## Results and Discussion

### General Remarks

In our previous work, CeO_2_ NPs and MnO_2_ MFs were prepared and thoroughly characterized.^15,39^ The hydrodynamic radius and zeta potential of CeO_2_ NPs are heavily dependent on the pH of the medium, with positively charged and negatively charged particles in solutions of acidic and alkaline pH, respectively. At pH 4.0, CeO_2_ NPs possess a hydrodynamic radius of 181 ± 24 nm and a zeta potential of 32 ± 1 mV. In alkaline conditions (pH 9.0), on the other hand, the negatively charged nanoceria have a hydrodynamic radius of 180 ± 31 nm and a zeta potential of −29 ± 1 mV. The IEP of CeO_2_ NPs dispersion (50 mg/L) occurred at a pH of 6.2. The dependence of the aggregation rate on the ionic strength was also pH-dependent, with a significant difference in the critical coagulation concentration (CCC), which is the ionic strength (in terms of NaCl concentration), after which the system aggregates rapidly. At pH 4.0, the CCC occurred at 79.1 mM and decreased by about 1 order of magnitude to 5.1 mM at pH 9.0.

The colloidal properties of MnO_2_ MFs also exhibited significant dependence on the pH. Acidic and neutral pH conditions resulted in rapid particle aggregation with low zeta potential, leading to the formation of micron-sized clusters. In increasingly alkaline conditions, a significant reduction of the particle size was observed. At pH 9.0, the recorded hydrodynamic radius and zeta potential of MnO_2_ MFs were 83 ± 2 nm and −37 ± 1 mV, respectively. In addition, the CCC of MnO_2_ MF dispersions (100 mg/L) at pH 9.0 occurred at 12.2 mM. indicating moderate colloidal stability of the particles.

### PDADMAC Functionalization of the Nanozymes

**Figure 1:**
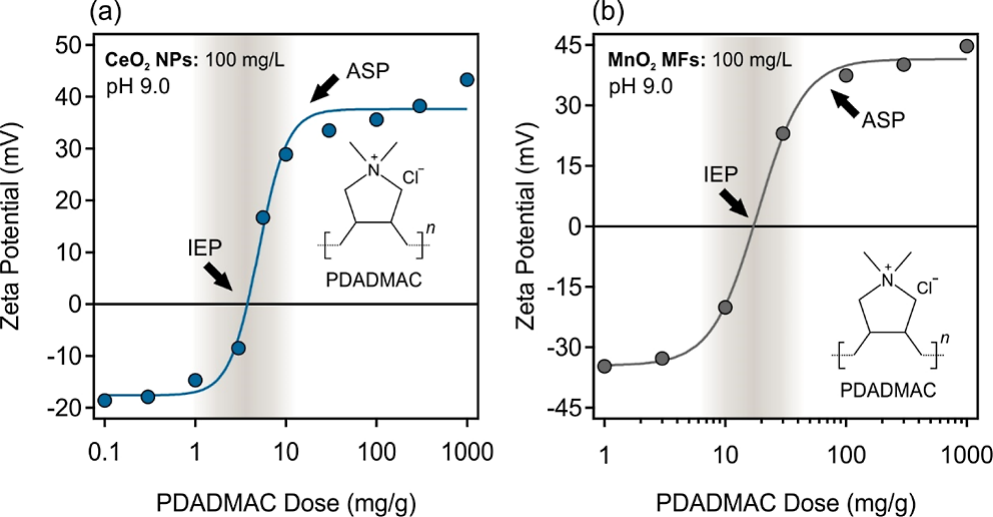
Zeta potential of PDADMAC-functionalized CeO_2_ NPs (a) and MnO_2_ MFs (b) at various PDADMAC doses (in mg PDADMAC/g metal oxide), pH 9.0, and 1.0 mM ionic strength. In both systems, the concentration of the metal oxide was fixed at 100 mg/L. Error bars for zeta potential data are too small to be visible as standard deviations occur in the range 0.1–1.4 mV.

Based on the above-discussed hydrodynamic radii and zeta potential values, the heteroaggregation of CeO_2_ NPs and MnO_2_ MFs can be carried out via electrostatic forces at pH 9.0 only if the surface charge is tuned since CeO_2_ NPs and MnO_2_ MFs are similarly charged. Hence, a positive charge must be induced on either component to enable their heteroaggregation through attractive forces of electrostatic nature. Accordingly, the functionalization of the metal oxides with the PDADMAC polyelectrolyte served to generate positively charged particles. As shown in Figure 1, variation in doses of PDADMAC (in mg PDADMAC/g metal oxide or mg/g) results in significantly different zeta potential values, enabling the control of the dominant interparticle forces and hence predicting the colloidal stability of the PDADMAC-functionalized metal oxides.

During the functionalization of both CeO_2_ NPs (Figure 1a) and MnO_2_ MFs (Figure 1b), low PDADMAC doses did not lead to changes in the zeta potential data, and thus, the particles remain negatively charged. However, an IEP was observed for both oxides, at which the positive and negative charges are balanced, resulting in functionalized nanozymes with no net charges. Literature data clearly indicate that if the zeta potential approaches zero near the IEP, the particles heavily aggregate under the influence of van der Waals forces.^55,59−61^ However, doses higher than those around the IEP induced charge reversal in both systems giving rise to positively charged metal oxide particles.

As shown in Figure 1a, the IEP of the PCe system occurred at a PDADMAC dose of 4.1 mg/g, while the dispersion of PMn exhibited an IEP at 17.2 mg/g. In addition, in both PCe and PMn dispersions, an adsorption saturation plateau (ASP) was identified at PDADMAC doses of 17.3 and 62.2 mg/g, respectively. Such an ASP value refers to the polyelectrolyte concentration, at which a saturated adsorbed layer forms and further added PDADMAC remains dissolved in solution.^55,61,62^ The dose selected for further heteroaggregation studies should give rise to highly charged particles, and thus, PDADMAC doses for PCe and PMn were 40 and 200 mg/g, respectively. These conditions are within the ASP, but the depletion attraction forces are avoided.^59^ Such polyelectrolyte functionalization proved as an effective way in modification of various surfaces and the charge neutralization, and reversal trend is well reported in the literature.^63,64^

### Heteroaggregation and Formation of Mn–PCe and Ce–PMn Hybrids

The heteroaggregation of Mn–PCe and Ce–PMn systems was achieved by co-assembly of the individual components at fixed pH (9.0) and ionic strength (1.0 mM). In each system, the concentration of the bare material (MnO_2_ MFs in Mn–PCe and CeO_2_ NPs in Ce–PMn) was fixed at 50 mg/L, while the dose of the PDADMAC-functionalized component (PCe in Mn–PCe and PMn in Ce–PMn) was varied to find the optimal experimental conditions to obtain stable colloids containing the different nanozymes in comparable amount.

**Figure 2:**
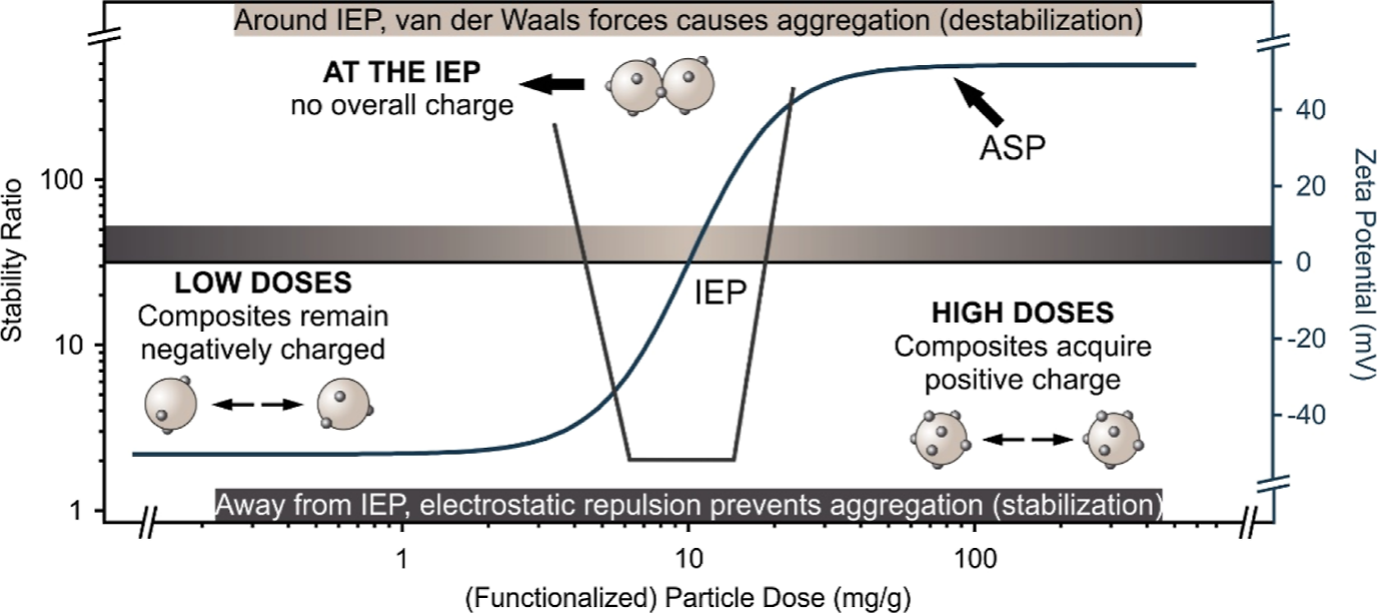
Anticipated trends in the stability ratio and zeta potential during heteroaggregation of oppositely charged particles, where the concentration of one component (bare CeO_2_ NPs or MnO_2_ MFs) is fixed, while the dose of the second component (PMO, either PCe or PMn, shown on the *x*-axis) is systematically varied.

Considering the theory developed by Derjaguin, Landau, Verwey, and Overbeek (DLVO)^60,65,66^ to describe the colloidal stability of particles, the expected trend in the stability ratio and zeta potential at various PMn or PCe doses is shown in Figure 2. When the dose of the PMO is low, the composites maintain high zeta potential (owing to the original negative change of the bare materials) and high (or not even measurable) stability ratios. With higher PMO doses, the magnitude of the zeta potential is expected to decrease and eventually approach zero at the system-specific IEP, where van der Waals forces induce rapid aggregation and hence, low stability ratios. Higher PMO doses are expected to result in charge reversal, with growing magnitude of zeta potential, as the PMO concentration increases. Eventually, the surfaces of the bare materials are saturated with PMO particles, and the change in the zeta potential data reaches a steady state at the ASP.

In our earlier studies, such trends were observed for the heteroaggregation between polystyrene latex particles and inorganic nanoparticles such as MnO_2_, CeO_2_, and Prussian blue.^15,39,67^ Other studies involved the heteroaggregation of oppositely charged polystyrene latex particles^66,68^ or P(MMA-*co*-BA) latex particles with layered double hydroxide (LDH) platelets.^69^ In these studies, the carrier particles, i.e., the latex particles, are highly charged, stable, and hence, the above trend in the change of zeta potential is expected upon adsorption of oppositely charged particles on its surface. Here, however, the bare CeO_2_ NPs and MnO_2_ NFs are not as colloidally stable as the latex particles since van der Waals forces are much stronger, and thus, particle aggregation likely occurs, which renders obtaining the trend in Figure 2 to be difficult.

**Figure 3:**
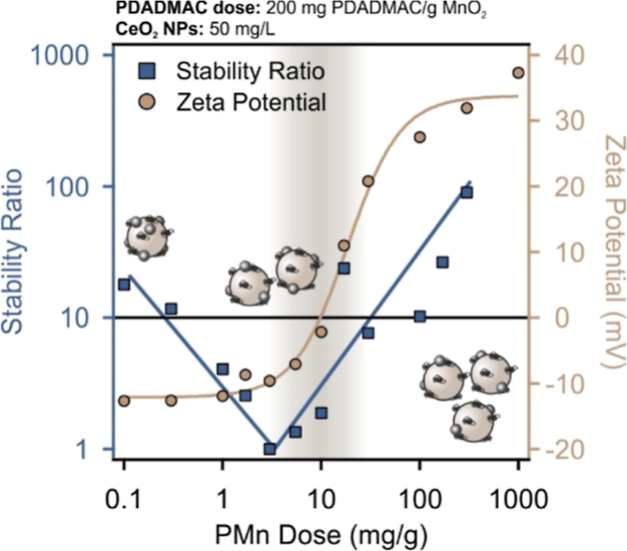
Stability ratio (squares) and zeta potential (circles) of the Ce–PMn system at various PMn doses (in mg PMn/g CeO_2_) and pH 9.0. The concentration of CeO_2_ NPs was fixed at 50 mg/L in all samples, while the ionic strength was set at 1.0 mM. The dose of PDADMAC in the pre-prepared PMn dispersion stock was set at 200 mg PDADMAC/g MnO_2_. Error bars of zeta potential values represent standard deviations occurring in the range of 0.1–1.4 mV, whereas the reproducibility of the stability ratio is established to be within 5% error.

The stability ratio and zeta potential data of the Ce–PMn system are shown in Figure 3. Unlike the trend in Figure 2, low PMn (at 200 mg PDADMAC/g MnO_2_) doses resulted in significantly lower zeta potential and stability ratio, indicating the moderate stability of the particles in this regime. Thereafter, the increase in the potentials by increasing the PMn dose is a clear sign of the formation of the Ce–PMn composites, leading to an IEP at a PMn dose of 9.8 mg/g. The system is characterized by stability ratios close to unity near the IEP, in line with the tendency in the interparticle forces explained in Figure 2. Higher doses led to charge reversal and eventual saturation near the onset of the ASP (around 200 mg/g). Beyond that dose, the hybrid Ce–PMn particles possess high zeta potential as well as high stability ratios, clearly indicating the formation of positively charged composites of remarkable dispersion stability.

**Figure 4:**
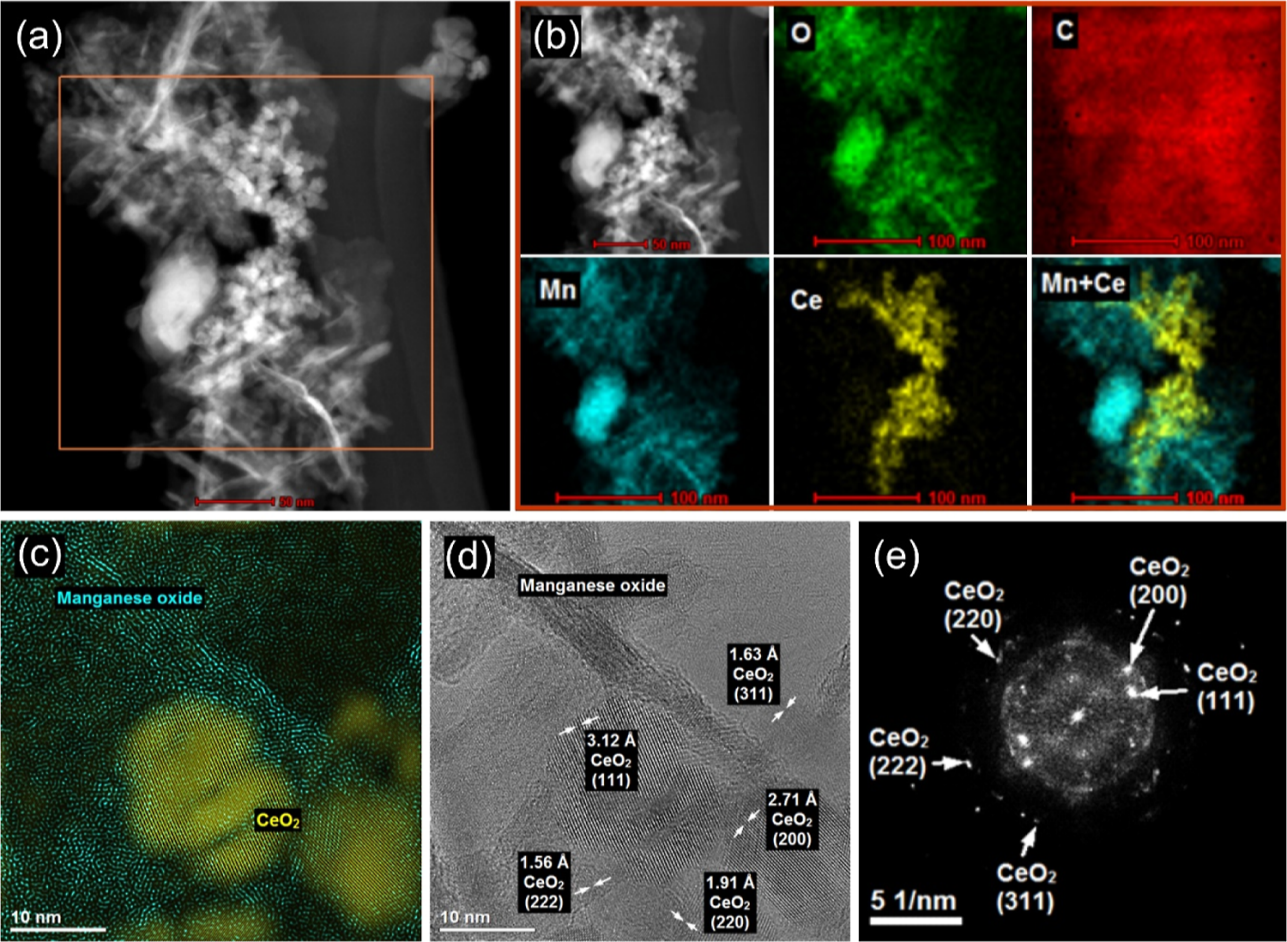
STEM-EDS mapping result of Ce–PMn (1000 mg/g). (a) STEM image of the composite shows the CeO_2_ NPs on the PMn phase. (b) Elemental mapping of the squared region in the STEM image in (a) for the elements O, C, Mn, and Ce. (c,d) HR-TEM images of Ce–PMn locating CeO_2_ NPs and MnO_2_ MFs as well as indexed lattice spacings. (e) FFT analysis with extraction patterns and the indexed lattice spacings, as obtained by inverse FFT analysis.

The Ce–PMn composite formulation was confirmed using STEM imaging. The STEM image in Figure 4a clearly presents the CeO_2_ NPs dispersed on the larger PMn particle. In addition, the elemental map in Figure 4b of the squared region in the STEM image shows the presence of O, C, Mn, and Ce. The presence of carbon can be attributed to the PDADMAC, as well as to the adventitious carbon on the surfaces of the metal oxides, as discussed in the previously reported surface analyses of these particles.^15,39^ The elemental mapping also shows that the composite is characterized by Ce-rich regions, rather than uniform distribution of CeO_2_ NPs onto PMn. In addition, the morphology of the composite was explored with HR-TEM. The HR-TEM images in Figure 4c,d indicate that CeO_2_ NPs are immobilized onto MnO_2_ MFs and that CeO_2_ NPs are highly crystalline with variously spaced crystal fringes. The FFT analysis was performed to obtain the extraction pattern (Figure 4e), from which the lattice spacings were obtained by inverse FFT analysis. These spacings are also presented in Figure 4d and were properly indexed to the corresponding XRD peaks reported elsewhere.^15,39^

**Figure 5:**
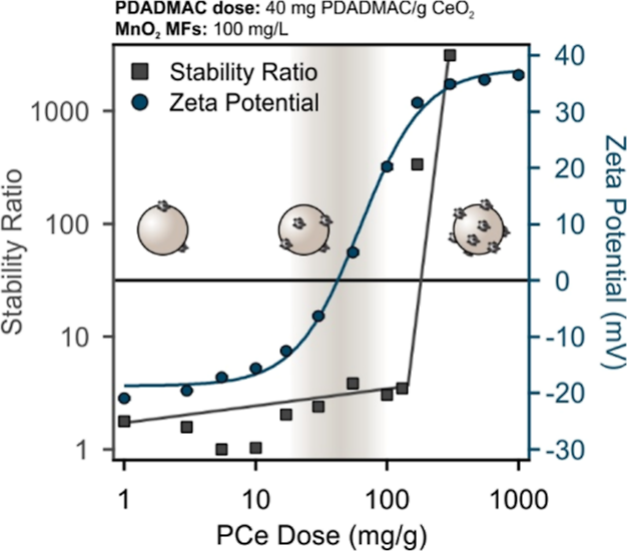
Trend in stability ratio (squares) and zeta potential (circles) of the Mn–PCe system at pH 9.0 and ionic strength of 1.0 mM. The MnO_2_ MFs concentration was set at 100 mg/L while the PCe doses (in mg PCe/g MnO_2_) were varied. The PDADMAC dose in PCe was set at 40 mg PDADMAC/g CeO_2_. The reproducibility of stability ratio is established to be within 5% error, while zeta potential measurements exhibited fluctuations in the range 0.1–1.4 mV.

Furthermore, the heteroaggregation of MnO_2_ MFs and PCe (at 40 mg PDADMAC/g CeO_2_) exhibited similar trends (Figure 5). The change in the stability ratio and zeta potential values was different from the one anticipated in Figure 2. Accordingly, low PMn doses resulted in a rather low zeta potential and stability ratio, indicating rapid particle aggregation in this regime. The Mn–PCe exhibited an IEP at a PCe dose of 42.5 mg/g, at which the stability ratio values remained low. The onset of the ASP occurred at PCe doses around 500 mg/g, where the composite particles were highly stable because of the presence of strong electrostatic repulsion. Based on these results, the stabilizing effect of the adsorbing PCe particles is obvious since bare or partially covered MnO_2_ NFs aggregated rapidly, while PCe decoration led to the formation of fine colloids containing highly stable Mn–PCe composites.

**Figure 6:**
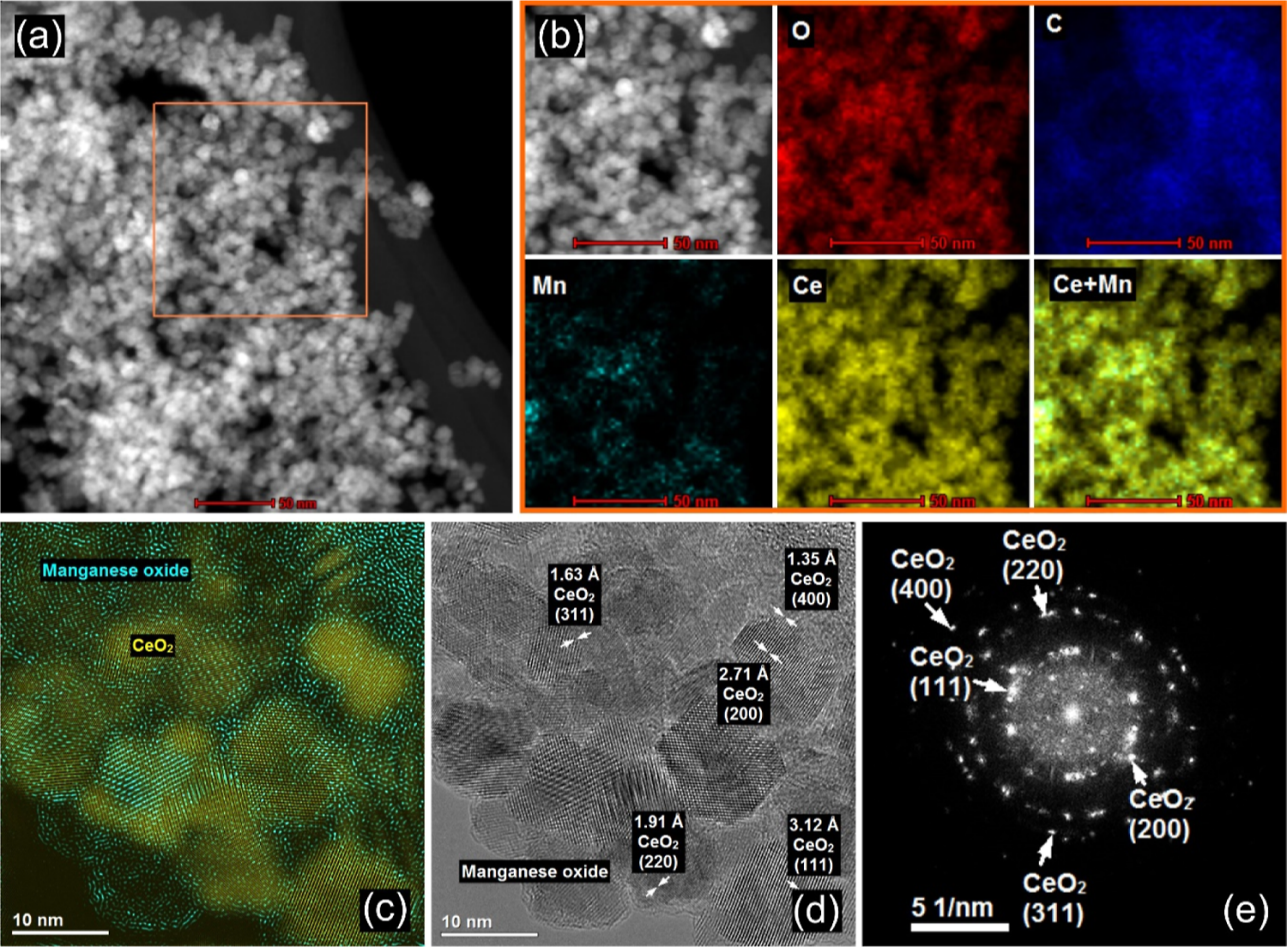
STEM-EDS mapping results of Mn–PCe (1000 mg/g) hybrids. (a) STEM image of the composite shows the PCe on the MnO_2_ MFs phase. (b) Elemental mapping of the squared region in the STEM image in (a) reveals the presence of O, C, Mn, and Ce. (c,d) HR-TEM images of Mn–PCe with elemental mapping of CeO_2_ NPs and MnO_2_ MFs, as well as indexed lattice spacings. (e) FFT analysis with extraction patterns and the indexed lattice spacings, as obtained by inverse FFT analysis.

The formation of the Mn–PCe composite was also explored by STEM imaging. Figure 6a represents the STEM image that clearly shows the PCe particles were located together with MnO_2_ MFs. In addition, the elemental distribution of the squared region in the STEM image (Figure 6b) indicates the presence of the elements O, C, Mn, and Ce. The results can be discussed in a similar fashion as for the Ce–PMn systems above.

Accordingly, the elemental mapping shows that the composite is characterized by Ce-rich regimes. In this combination, the PCe particles were immobilized onto PMn to a much larger extent compared to the case, when bare CeO_2_ NPs were heteroaggregated with PMn. The results of further enzyme activity measurements may also reflect such a difference in the abundance of the ceria in the composites. Besides, HR-TEM images in Figure 6c,d unambiguously confirms that CeO_2_ NPs are distributed onto MnO_2_ MFs. The crystal fringes of the ceria nanozymes (Figure 6e), as well as the inverse FFT analysis gave rise to conclusions resembling those drawn during the discussion of the Ce–PMn systems.

In general, once the doses of the PDADMAC-functionalized particles were gradually increased, charge neutralization and reversal were observed, which provide control over the colloidal stability dominated mainly by DLVO forces, which have various contributions at different experimental conditions. Hence, the above results are significant, as controlling and fine tuning the naturally aggregating particles is essential in many applications. In fact, composites prepared by heteroaggregation by simple mixing of dispersions of the components have been widely utilized in various fields including coating, food packaging, anti-microbial agents, (photo)catalysis, environmental remediation including wastewater treatment, optimal and bioactive materials, as well as electrochemical devices such as sensors, solar cells, batteries, and capacitors.^50^ Nevertheless, the above results were exploited to study possible synergistic or complementary effects of the nanozymes in antioxidant assays.

### Antioxidant Activity

The SOD-like activity is typically assessed using the Fridovich assay,^56^ which relates the extent of the inhibition of the NBT reduction with the superoxide radical ion scavenging potential of the composites. The inhibition of the superoxide radical-NBT reaction, obtained using eq 4, is reported at the corresponding antioxidant concentration.

**Figure 7:**
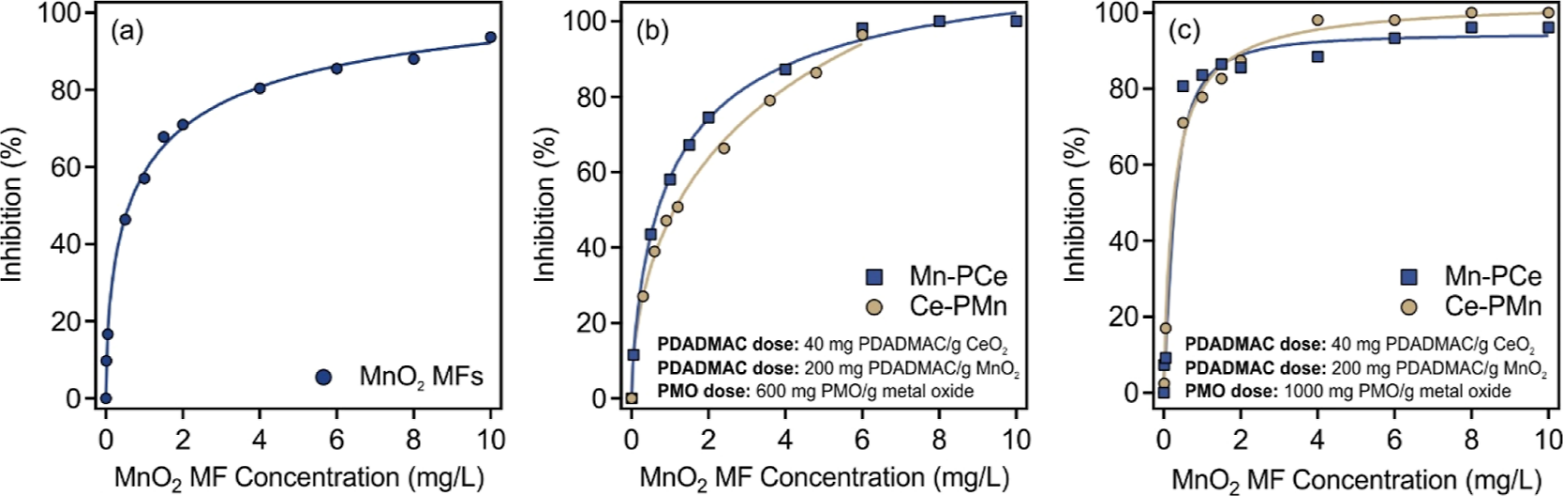
SOD-like activity (inhibition of NBT reduction) of (a) bare MnO_2_ MFs, (b) Mn–PCe and Ce–PMn (600 mg/g), and (c) Mn–PCe and Ce–PMn (1000 mg/g).

As shown in Figure 7a, bare MnO_2_ MFs exhibited excellent SOD-like activity, and higher MnO_2_ MF concentration resulted in greater inhibition, i.e., radical scavenging, which approached 100% at a MnO_2_ MF concentration of about 10 mg/L. The bare CeO_2_ NPs did not possess SOD-like features, possibly due to the low Ce^3+^/Ce^4+^ ratio on the surface, as reported in our previous study.^39^

In addition, the activity of the composites (Ce–PMn and Mn–PCe) was assessed at two different PMO doses to probe the possibility of synergetic effects between the two nanozymes. Figure 7b,c shows the inhibition for the two composites at 600 and 1000 mg/g (in mg PMO/g metal oxide) concentrations, respectively. At a given MnO_2_ MFs or PMn concentration, the SOD-like function was not significantly affected by the presence of CeO_2_ NPs or PCe, and the difference remains within the experimental error of the assay method (∼10%). This indicates that there was no significant interaction between the two components and that the MnO_2_ MFs operate independently as a SOD mimicking material. The IC_50_ value of the MnO_2_ MFs was 0.60 mg/L, which did not change significantly upon heteroaggregation with CeO_2_ NPs. This is higher than that of the native SOD, which is reported to be 0.07 mg/L.^70^ Although it is superior in activity compared to those of the composites, the colloidal and functional stability of the hybrids, as well as the ease and low cost of preparation outweigh the lower superoxide radical scavenging ability. In fact, the SOD enzyme loses activity after 20 min of incubation at 80 °C,^71^ while the SOD-like function of MnO_2_ MFs is not changed even after thermal treatment for 90 min at 75 °C.^15^

**Figure 8:**
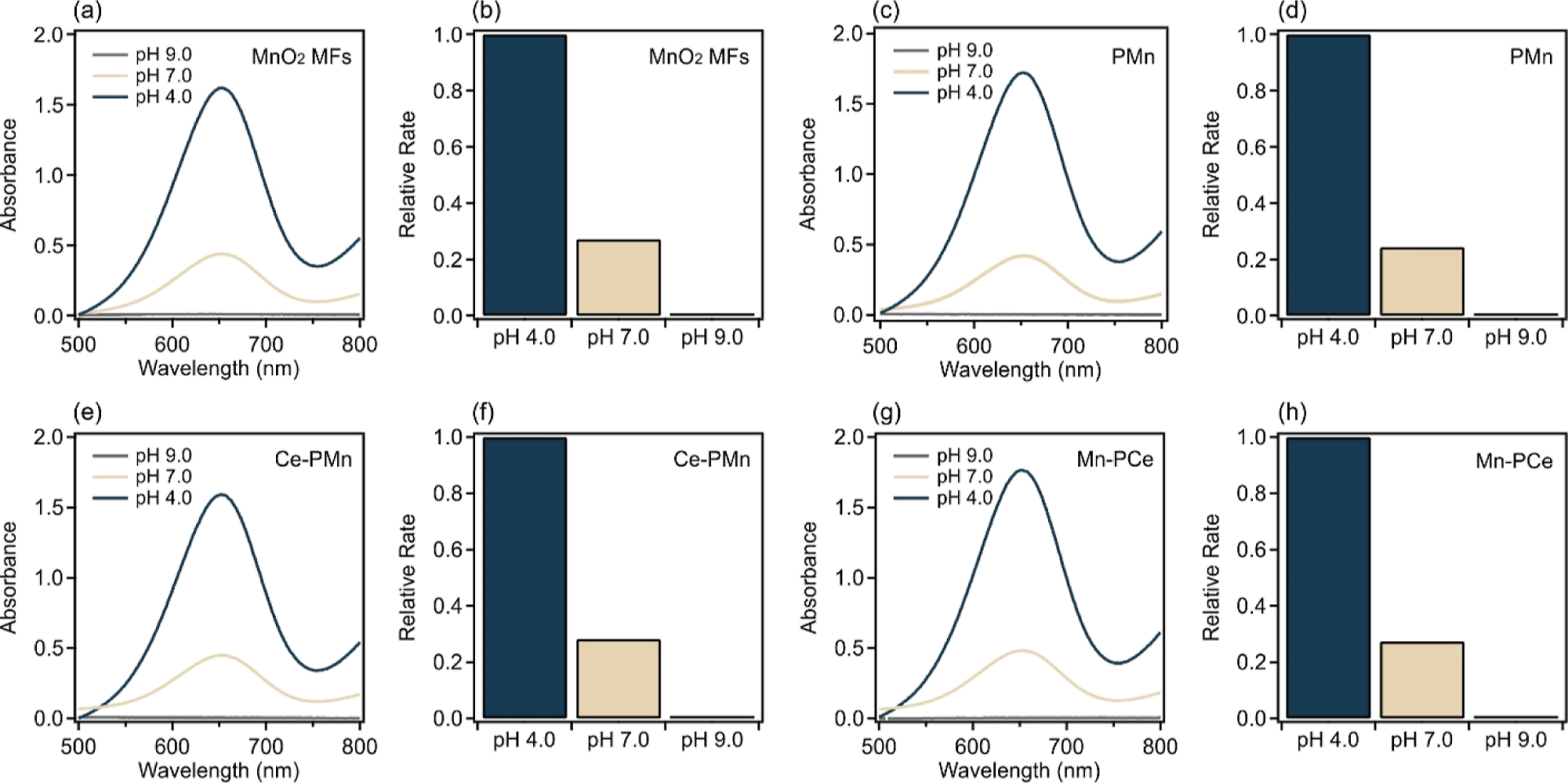
Oxidase-like activity of (a,b) MnO_2_ MFs, (c,d) PMn, (e,f) Ce–PMn, and (g,h) Mn–PCe composite. In each sample, relevant concentrations are 0.5 mM TMB and 15.0 mg/L MnO_2_ MFs or PMn. The dose of PMO in both composites is 1000 mg/g. The acidic, neutral, and alkaline pH conditions were set using acetate, phosphate, and Tris buffers (50 mM), respectively. The spectra of samples were recorded after the enzymatic reaction. The relative rate was obtained by comparing the absorbance values at 652 nm.

The oxidase activity of the bare and composite materials was examined using TMB as a substrate, which is directly oxidized by the oxidase mimicking nanozymes into a blue product with a strong absorption peak at 652 nm.^58^Figure 8 shows the emergence of such a peak at different pH conditions for the different materials.

Figure 8a shows that MnO_2_ MFs develop a strong peak at 652 nm under acidic conditions, indicating a strong oxidase mimicking ability that was observed instantaneously upon mixing the relevant components. At pH 7.0, a decreased activity was observed, as indicated by the lower absorbance value. In addition, no oxidase-type function was observed at pH 9.0 since the oxidation of TMB requires at least slightly acidic media. The relative rate of TMB oxidation at different pH conditions is shown in Figure 8b. Similarly, the oxidase-like behavior of PMn is presented in Figure 8c,d with higher activity in more acidic and no operation in alkaline media. The PDADMAC functionalization did not affect the activity since similar absorbance values were obtained. The CeO_2_ NPs did not exhibit any oxidase functions,^39^ and hence, the data measured for Ce–PMn and Mn–PCe are expected to resemble those of MnO_2_ MFs, if no interactions occurred between the two metal oxides, which is clearly observed in Figure 8e–h.

**Figure 9:**
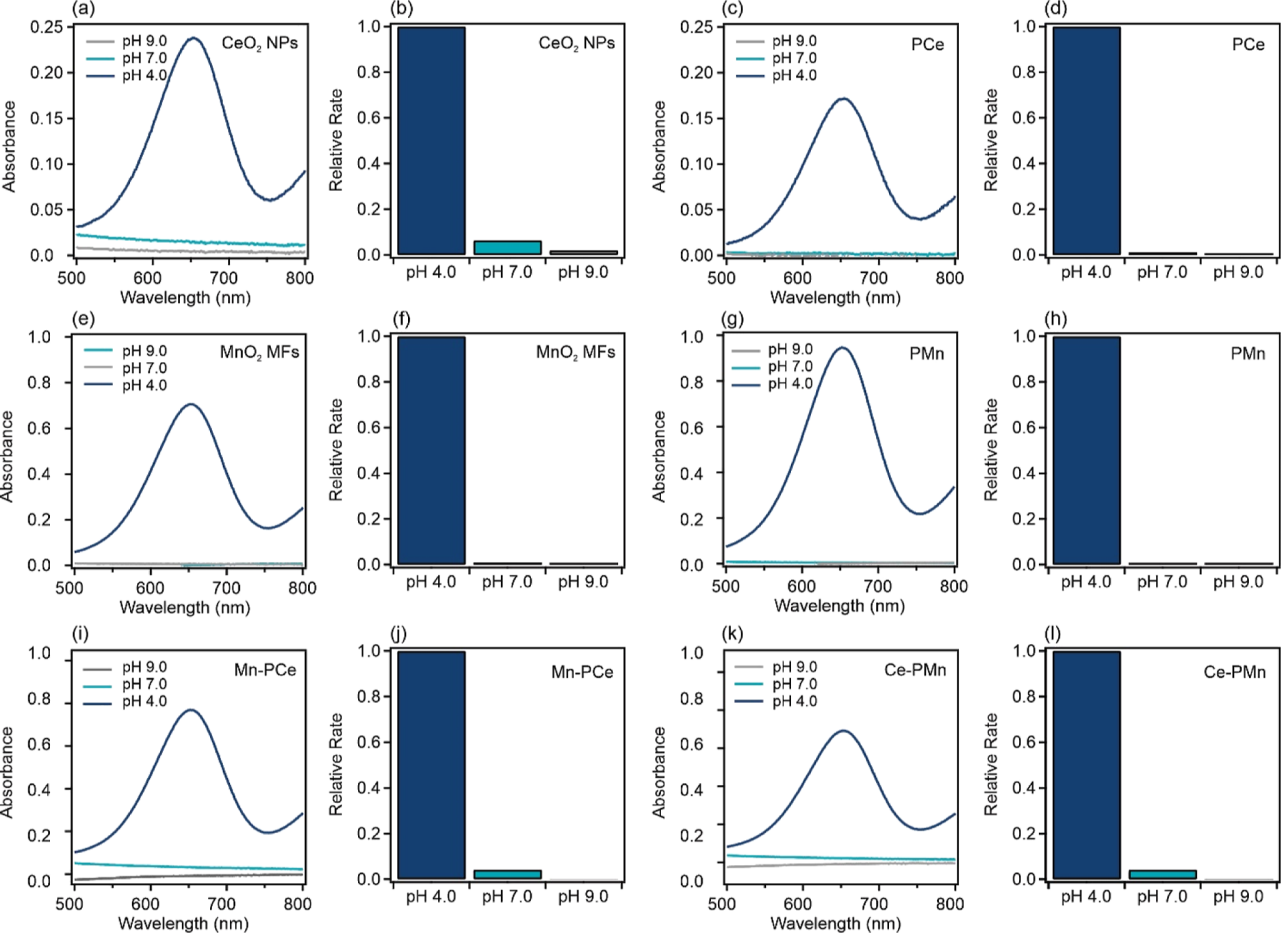
Peroxidase-like activity of (a,b) CeO_2_ NPs, (c,d) PCe, (e,f) MnO_2_ MFs, (g,h) PMn, (i,k) Mn–PCe, and (k,l) Ce–PMn. In each sample, relevant concentrations are 15.0 mM H_2_O_2_, 0.5 mM TMB, and 15.0 mg/L nanozymes (or bare nanozymes in the case of the composite). The dose of PMO in both composites is 1000 mg/g. The acidic, neutral, and alkaline pH conditions were set using acetate, phosphate, and TRIS buffers (50 mM), respectively. The spectra were recorded after the enzymatic reaction terminated. The relative rate was obtained by comparing the absorbance values at 652 nm.

Similarly, the peroxidase mimicking activity of the bare and composite materials was examined using TMB assay as well. Native peroxidase, or its mimicking equivalent, breaks down H_2_O_2_ into intermediate radicals that further oxidize the TMB, resulting in a blue solution and an absorption peak at 652 nm.^57^ As shown in Figure 9a–d, the evolution of the peak of the oxidized TMB is observed in the presence of CeO_2_ NPs or PCe, as well as H_2_O_2_. The peroxidase-type function was detected under acidic conditions (pH 4.0), while no activity was observed under neutral and alkaline conditions.

On the other hand, since MnO_2_ MFs exhibited oxidase-like operation, it is quite difficult to probe the peroxidase-like activity of MnO_2_ MFs or MnO_2_-containing composites since both activities will contribute to the oxidation of TMB. Figure 9e–h shows the evolution of the absorbance of TMB (0.5 mM) at 652 nm in the presence of 15 mg/L MnO_2_ MFs (or PMn) and 15.0 mM H_2_O_2_. Within the same timeframe and at the same pH, the total absorbance of TMB is less than that for the oxidase activity (Figure 8a). Note that the catalase function (breakdown of H_2_O_2_) of MnO_2_ MFs was established in our earlier work.^15^ Hence, the oxidase activity is diminished by the simultaneous catalase mimicking ability, resulting in decreased direct TMB oxidation since a proportion of the catalytic surface is occupied with catalase activity. For the Mn–PCe (Figure 9i,j) and Ce–PMn (Figure 9k,l) composites, the total absorbance comprises the oxidase and peroxidase of MnO_2_ MFs and CeO_2_ NPs, respectively. In both cases, the activity is only observed under acidic conditions.

Based on these results, one can notice that the obtained composites possess multiple enzymatic activities, i.e., SOD, oxidase, and peroxidase functions were all observed, due to the complementary activities of the individual components. Therefore, such hybrid materials can be used as broad-spectrum antioxidants, especially in colloidal systems owing to their remarkable dispersion stability.

## Conclusions

A thorough investigation of the formation of two antioxidant composites (Mn–PCe and Ce–PMn) is presented. Previously characterized MnO_2_ MFs and CeO_2_ NPs were formulated into composites by PDADMAC adsorption and subsequent heteroaggregation of properly selected mass ratios. The adsorption of PDADMAC on the negatively charged metal oxides enabled heteroaggregation via electrostatic attraction between the bare and polyelectrolyte-functionalized particles. The variation in the dose of the PMO gave rise to negatively, neutral, and positively charged hybrids as well as systems with varying contributions of the DLVO-type forces. At high doses of PMO, the hybrid particles have high overall charge, and hence, the dispersions are stabilized by electrostatic repulsion. At doses close to the system-specific IEP values, the composites possess no overall charge, and hence, the dispersion is characterized by rapid aggregation under the influence of van der Waals forces. The formulated composites exhibited SOD, oxidase, and peroxidase-like activities. Although the activity of these composites might not be comparable to the native enzymes, the associated benefits outweigh the reduction in the activities. Such advantages include lower production costs, simpler preparation/storage, as well as controllable colloidal properties, which were tailored by carefully selecting the mass ratios. The present study demonstrates and paves the way for the formation of more complex composite structures by a simple control and understanding of the colloidal properties resulting in antioxidant composites with potential use in various industrial applications.
